# Preliminary insights regarding the quality of Kallmet wine, obtained by sequential inoculation with *Metschnikowia pulcherrima* and *Saccharomyces cerevisiae*

**DOI:** 10.3389/fmicb.2025.1654308

**Published:** 2025-08-26

**Authors:** Mamica Ruci, Renata Kongoli, Francesca Coppola, Mariantonietta Succi, Bruno Testa, Onejda Kyçyk, Julian Karaulli, Fatbardha Lamçe, Massimo Iorizzo

**Affiliations:** ^1^Faculty of Biotechnology and Food, Food Research Center, Agricultural University of Tirana, Tirana, Albania; ^2^Department of Agro-food Technology, Faculty of Biotechnology and Food, Agricultural University of Tirana, Tirana, Albania; ^3^Department of Agricultural Sciences, University of Naples “Federico II”, Portici, Italy; ^4^Department of Agriculture, Environmental and Food Sciences, University of Molise, Campobasso, Italy

**Keywords:** non-*Saccharomyce*s, *Metschnikowia pulcherrima*, inoculation timing, Kallmet wine, sensorial analysis, VOCs

## Abstract

Non-*Saccharomyces* wine yeasts have a promising role in biotechnological approaches to enhance wine complexity, particularly by influencing the aromatic profile. *Metschnikowia pulcherrima* is a non-*Saccharomyces* yeast that is notable for its antimicrobial activity and diverse enzymatic activities. These properties make this yeast a valid candidate for application as a starter culture in winemaking. This study evaluated the application of *M. pulcherrima 62* in sequential inoculation with *S. cerevisiae* for the production of Kallmet wine, delivered from the indigenous Kallmet grape variety traditionally cultivated in various regions of Albania. The use of different inoculation strategies resulted in significant differences in wine composition, affecting key oenological parameters, the aromatic profile and sensory attributes. *M. pulcherrima* 62 did not negatively interfere with the fermentation kinetics of *S. cerevisiae* during fermentation and contributed to wines with lower amounts of ethanol and richer in glycerol, total polyphenols and anthocyanins. Moreover, increased levels of isobutanol, phenylethyl alcohol, isoamyl alcohol and monoterpenes (linalool, geraniol, and nerol) were observed in wines produced with *M. pulcherrima* 62 and *S. cerevisiae* in sequential inoculation, compared to wines fermented solely with *S. cerevisiae*. Finally, sensory analysis revealed a distinct differentiation in the wines, attributable to the modulation of aromatic compounds by *M. pulcherrima* 62.

## 1 Introduction

During alcoholic fermentation, yeasts primarily convert sugars like glucose and fructose into ethanol and carbon dioxide for energy production and cell growth. This process, known as primary metabolism, is crucial for yeast survival and fermentation efficiency. In addition to this, yeasts also carry out a secondary metabolism which involves the production of a wide range of byproducts - such as esters, higher alcohols and organic acids—that play a significant role in defining the flavor and aroma of fermented beverages (Comitini et al., 2021; Romano et al., 2022; Maicas and Mateo, 2023).

Traditionally, *Saccharomyces cerevisiae* has been the dominant species used in winemaking due to its ability to efficiently ferment grape sugars, withstand harsh winemaking conditions and produce desirable sensory profile (Parapouli et al., 2020).

However, in recent years, the controlled use of non-*Saccharomyces* yeasts in winemaking has grown enormously, quickly becoming a biotechnological tool to improve the chemical and sensory properties of wine (Borren and Tian, 2020; Perpetuini et al., 2020; Tofalo et al., 2022). In particular, mixed-culture fermentations involving selected non-*Saccharomyces* and *Saccharomyces* strains have gained attention in recent years due to their ability to modulate a wide range of metabolites of oenological interest (Tofalo et al., 2016; Lombardi et al., 2018; Testa et al., 2020, 2021). Generally, in mixed-culture fermentations, the distinctive enzymatic activities of non-*Saccharomyces* yeasts contribute to the production of aromas and flavors that cannot be obtained using *S. cerevisiae* alone as a single starter (Englezos et al., 2022).

In this context, the use of selected non-*Saccharomyces* yeasts in the fermentation process has been proposed as a novel strategy to reduce the ethanol content and enhance the sensory characteristics of wine (Ciani et al., 2010; Testa et al., 2021). Among non-*Saccharomyces* yeasts, *Metschnikowia pulcherrima*, has garnered considerable oenological interest due to its remarkable properties.

Compared to *S. cerevisiae*, *M. pulcherrima* has a lower fermentative capacity and can be used in winemaking to produce wines with reduced ethanol content, a growing trend in the industry (Canonico et al., 2019a). In addition, *M. pulcherrima* contributes to the aromatic profile of wine through the production of various metabolites such as esters and higher alcohols facilitated by its enzymatic activities particularly *β*-glucosidase and β-lyase (Torres-Díaz et al., 2024).

In addition, hydrolytic enzymes of *M. pulcherrima*, such as protease and polygalacturonase, facilitate the extraction of color and aroma precursors from grapes (Testa et al., 2024). Proteases degrade proteins, while polygalacturonase, also known as pectinase, breaks down pectin. This helps to release natural flavors and pigments from the grapes, which contribute to the aroma and color of wines (Chen et al., 2018).

Recent studies have shown that *M. pulcherrima* acts as a biocontrol agent against wine spoilage microorganisms, potentially reducing the need for chemical preservatives (Simonin et al., 2020; Aragno et al., 2024).

*M. pulcherrima* has a high capacity for oxygen consumption (Canonico et al., 2019a; Chacon-Rodriguez et al., 2020). The reduction of oxygen in the must helps inhibit the growth of other microorganisms (Windholtz et al., 2021; Di Gianvito et al., 2022) and significantly decreases the substrate necessary for the oxidative action of polyphenoloxidases (Giménez et al., 2023).

Therefore, the use of *M. pulcherrima* as a starter culture is particularly relevant in winemaking for preventing browning of white grape musts (Giménez et al., 2023; Bustamante et al., 2024).

The proteolytic activity of *M. pulcherrima* is a key feature, particularly when used in co-fermentation with other yeasts. In this regard, it facilitates the release of amino acids that serve as nutrients for *S. cerevisiae*, while also acting as a biological fining agent to control protein haze formation in wine (Marangon et al., 2012; Hong et al., 2019).

Due to the properties described above, *M. pulcherrima* is increasingly offered as a starter culture in winemaking (Vicente et al., 2020; Perpetuini et al., 2023; Testa et al., 2024). Table 1 provides an overview of studies investigating the use of *M. pulcherrima* in sequential inoculation with *S. cerevisiae* for the fermentation of musts obtained from different grape varieties. However, to date, few *M. pulcherrima* strains available on the market are specifically promoted for their antimicrobial activity and their ability to enhance wine’s aromatic complexity (Coppola et al., 2025).

**Table 1:** Overview of the key results achieved through the sequential inoculation of *M. pulcherrima* with *S. cerevisiae* in the vinification of different grape varieties.

Starter cultures	Grape variety	Main effects in wine	References
*M. pulcherrima* AS3C1/ *S. cerevisiae* Actiflore® F33	Aglianico	↑ anthocyanins, catechins and color intensity; ↓ volatile acidity	Testa et al. (2024)
*M. pulcherrima* DiSVA269/ *S. cerevisiae* AWRI 838	Chardonnay	↓ ethanol; ↑ glycerol and esters	Canonico et al. (2019b)
*M. pulcherrima* AWRI1149/ *S. cerevisiae* AWRI1631	Chardonnay and Shiraz	↑ esters, higher alcohols and glycerol; ↓ ethanol	Varela et al. (2016)
*M. pulcherrima* AS3C1/ *S. cerevisiae* ENOFERM T306	Falanghina	↑ esters and higher alcohols; ↑ sensory profile	Coppola et al. (2025)
*M. pulcherrima* CLI 68 and *M. pulcherrima* CLI 460/ *S. cerevisiae* CLI 889	Malvar	↓ ethanol; ↑ glycerol, higher alcohols and esters	García et al. (2020)
*M. pulcherrima* CECT12841/ *S. cerevisiae* EC1118	Malvasia and Viura	↑ glycerol; ↓ ethanol and acetic acid	Morales et al. (2015)
*M. pulcherrima* P01A016/ *S. cerevisiae* Enoferm Syrah	Merlot	↓ ethanol	Aplin et al. (2021)
*M. pulcherrima* GS80/ *S. cerevisiae* SRS1	Pecorino	↑ esters and terpenes; ↓ ethanol; ↓ titratable and volatile acidity	Perpetuini et al. (2023)
*M. pulcherrima* MCR-24*/ S. cerevisiae* PB2023	Sauvignon blanc	↑ higher alcohols, terpenols and esters ↓ ethanol and acetic acid	Sadoudi et al. (2012)
*M. pulcherrima* Lc3LT30/ *S. cerevisiae Zymaflore* X5	Sauvignon blanc	↑ thiols	Zott et al. (2011)
*M. pulcherrima* AWRI1149/ *S. cerevisiae* AWRI1631	Shiraz	↓ ethanol and acetic acid	Contreras et al. (2015)
*M. pulcherrima* Y0839/ *S. cerevisiae* VIN13 and *S. cerevisiae* NT202	Syrah	↓ alcohol and volatile acidity	Minnaar et al. (2019)
*M. pulcherrima* ARC/ *S. cerevisiae* VIN 13	Syrah	↑ flavonols and anthocyanins	Minnaar et al. (2017)
*M. pulcherrima* LAMAP-USACH L1781/ *S. cerevisiae*	Tempranillo	↑ esters and higher alcohols; ↑ total polyphenol	Escott et al. (2018)
*M. pulcherrima* 28 and 29/ *S. cerevisiae* VRB Lallemand	Tempranillo	↑ color	Escribano-Viana et al. (2019)
*M. pulcherrima* M28 and M29/ *S. cerevisiae* VRB	Tempranillo	↑ glycerol; ↓ volatile acidity	Escribano-Viana et al. (2018)
*M. pulcherrima* Mp39*/ S. cerevisiae* UCD522	Tinta Roriz	↓ ethanol, acetic acid and hydrogen sulfide	Barbosa et al. (2018)
*M. pulcherrima* NS-EM-34/ *S. cerevisiae* Viniferm Diana and *S. cerevisiae* Revelacion	Verdejo	↑ thiols	Ruiz et al. (2018)
*M. pulcherrima* DiSVA 269*/ S. cerevisiae* DiSVA 708 and *S. cerevisiae* Lalvin OKAY	Verdicchio	biocontrol; ↑ esters and higher alcohols	Canonico et al. (2023)
*M. pulcherrima* DiSVA 269/ *S. cerevisiae* Lalvin EC1118	Verdicchio	↓ ethanol and acetaldehyde; ↑ higher alcohols and esters	Canonico et al. (2019a)
*M. pulcherrima* DiSVA 269/ *S. cerevisiae* indigenous strains	Verdicchio	biocontrol; ↑ acetaldehyde, higher alcohols, esters and terpenes	Agarbati et al. (2023)
*M. pulcherrima* CVE-MP20/ *S. cerevisiae* SC45	Vidal	↓ ethanol and acetic acid ↑ esters and higher alcohols;	Zhang et al. (2018b)

During the last decade, Albania has seen a significant increase in wine production, which can be attributed to the growing interest in the diversity of native grape varieties. The Kallmet grape is an ancient Albanian cultivar that originates in the village of Kallmet and has historically been grown in the regions of Lezha and Shkodra, typically planted on hilly and sloping soils with sandy structures. Kallmet grapes have medium-sized, spherical deep red to violet berries and occupy about 20% of the vineyard area in Albania (Susaj et al., 2012).

Kallmet wine hold a Geographical Indication (GI) in Albania and is distinguished by its high content of flavonoids and stilbenes (Kullaj and Çakalli, 2013; Morina and Kongoli, 2014; Peçuli et al., 2018; Topi et al., 2024).

In recent years, Kallmet wines have significantly increased in value, representing an attractive and high-quality alternative to other wines with GI status in Albania (Caso and Giordano, 2022). The aim of this study was to evaluate the impact of *M. pulcherrima* 62, isolated from a vineyard (Karaulli et al., 2023), in the winemaking of Kallmet wine.

## 2 Materials and methods

### 2.1 Yeast strains and growth conditions

In this study, *M. pulcherrima* 62 (GenBank accession number: PP922568), belonging to the culture collection of the Agri-Food Research Centre at the Faculty of Biotechnology and Food of the Agriculture University of Tirana, was used. This strain was previously isolated from autochthonous Albanian red grapes and thoroughly characterized (Karaulli et al., 2024). For the pilot-scale winemaking, the commercial strain *S. cerevisiae* Zymaflore F15 (Laffort, Bordeaux, France) was used as a reference. Prior to use, yeast strains were aerobically cultured in YEPD broth (Merck Millipore, Darmstadt, Germany) at 28°C. After 48 h of incubation, cultures were centrifuged at 5000 rpm for 10 min at 4°C. The resulting cell pellets were washed twice with 0.9% NaCl saline solution. Cell density of the inoculum (10^6^ CFU/mL) was determined using a Thoma Counting Chamber (Thermo Fisher Scientific, Waltham, MA, USA).

### 2.2 Winemaking process

In winemaking trials, Kallmet grapes (*Vitis vinifera* cv.) from 2018 vintage were used. The grapes were harvested and transported to the Agri-Food Research Centre at the Faculty of Biotechnology and Food, Agriculture University of Tirana. The grapes were destemmed and crushed without the addition of any adjuvants. The resulting grape must, showed the following chemical composition: pH 3.40, sugar 230 g/L, total acidity 7.50 g/L, and YAN (yeast assimilable nitrogen) 156 mg/*L. prior* to fermentation, 40 mg/L of potassium metabisulphite (Essedielle srl, Italy) was added to the must.

Three fermentation tests were conducted: Test A involved sequential inoculation with *M. pulcherrima* 62, followed by *S. cerevisiae* F15 48 h after the start of the trial; Test B followed the same procedure but with *S. cerevisiae* F15 added after 72 h; and Test C (control) was inoculated only with *S. cerevisiae* F15.

**Figure 1:**
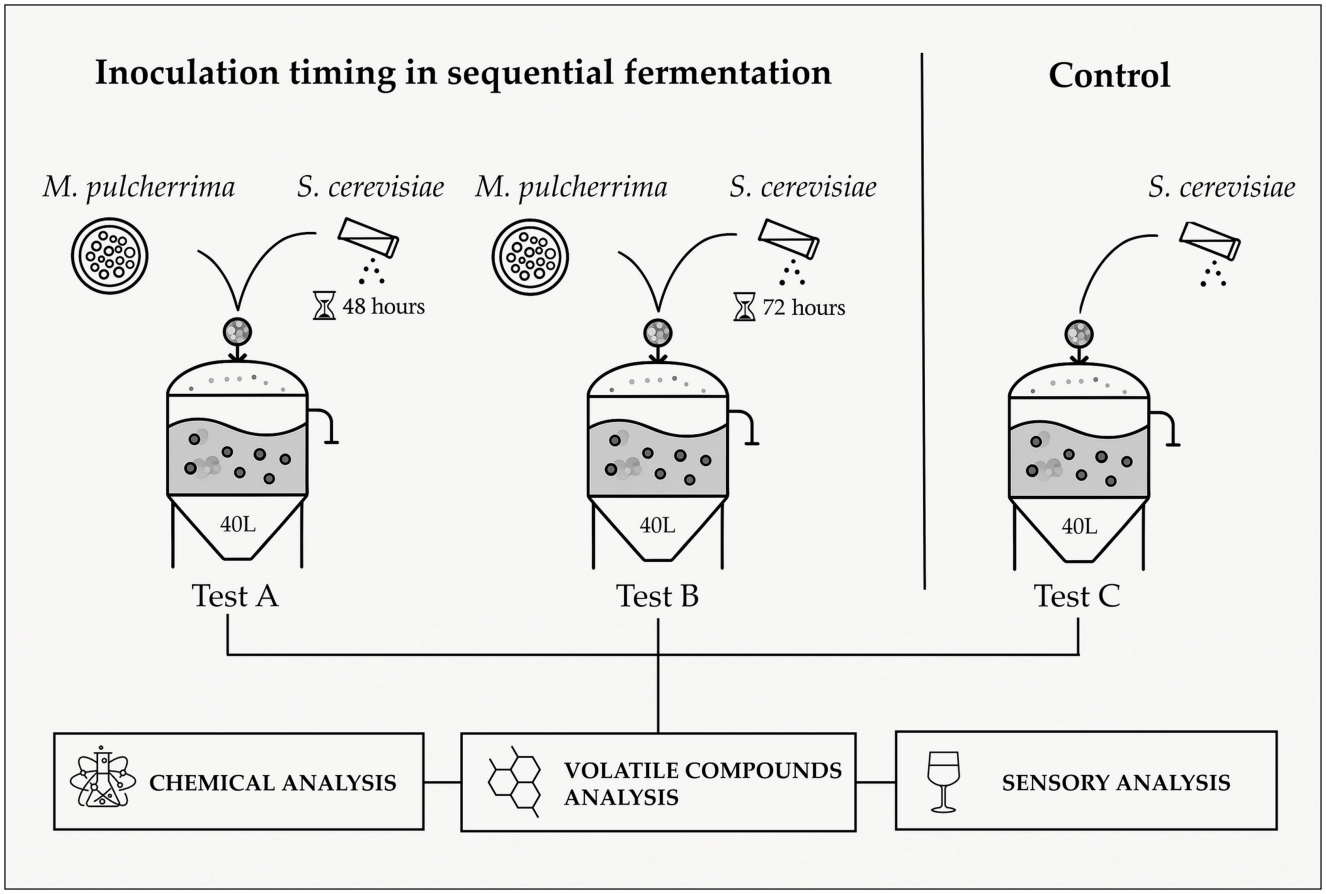
Experimental flowchart for the production of Kallmet wine utilizing different sequential inoculation timings of *M. pulcherrima* and *S. cerevisiae.*

Each test was carried out in triplicate in stainless steel tanks (working volume 50 L) containing 40 L of grape must with skins. The experimental flowchart illustrating the different sequential inoculation timings used to produce Kallmet wines is shown in Figure 1. Starter yeasts were inoculated to achieve an initial cell density of approximately 10^6^ CFU/mL. Fermentation was carried out at a controlled temperature of 24°C (± 2°C).

### 2.3 Fermentation process monitoring

Fermentation kinetics were monitored by assessing the ethanol production and yeast viability. Viable yeast cell counts were determined using WL agar (Merck KGaA, Darmstadt, Germany) supplemented with 100 mg/L chloramphenicol to inhibit bacterial growth. Plates were incubated aerobically at 28°C for 72 h. Colony color and morphology were used to differentiate *S. cerevisiae*, *M. pulcherrima*, and other yeast species (Pallmann et al., 2001). Yeast species identification was confirmed by molecular analysis through sequencing of the D1/D2 domain of the 26S rDNA gene (Testa et al., 2024).

### 2.4 Chemical analysis

At the end of alcoholic fermentation, the main chemical parameters of the wines were determined. The pH (OIV-MA-AS313-15), alcohol content % v/v (OIV-MA-AS312-01B), total acidity g/L as tartaric acid (OIV-MA-AS313-01), volatile acidity g/L as acetic acid, (OIV-MA-AS313-02) and reducing sugar g/L (OIV-MA-AS311-02), were measured according to OIV methods (OIV, 2011). L-malic acid (g/L), glycerol (g/L), anthocyanins (mg/L), total polyphenols (mg/L as gallic acid) and acetaldehydes (mg/L) were determined using enzymatic and colorimetric kits (Steroglass, Perugia, Italy), following the manufacturer’s instructions.

### 2.5 Volatile compounds analysis

The determination of major volatile compounds (VOCs) was carried out using gas chromatography (GC) according to OIV-MA-AS315-27 method (ET and VIN, 2011),. The instrument used (Thermoquest Mod. 8,000, Rodano, Milan, Italy) was equipped with a fused capillary column ZB-Wax (30 m × 0.32 mm i.d., 0.50 μm film thickness, Phenomenex, Torrance, CA, USA) and a flame ionization detector. Briefly, after the addition of an internal standard (butan-2-ol; 0.1 mg/mL in water), 1 μL of the sample was injected directly in split mode (1:50); injection port at 250°C. The oven temperature was increased from 40°C (held for 5 min) to 240°C at a rate of 7°C/min. Helium was used as the carrier gas at a flow rate of 60 kPa. All reagents were obtained from Merck Life Science (Milano).

### 2.6 Sensory analysis

Sensory evaluation of the wines was conducted by a trained panel of 20 judges (10 females, 10 males), aged 20–60, recruited from the National Organization of Wine Tasters (ONAV, Italy). Sensory assessments were performed in three sessions, during each of which panelists evaluated three wines in randomized order. Samples (30 mL) were served at 18°C in black tulip-shaped glasses, covered with glass Petri dishes and labeled with randomized three-digit codes. Unsalted crackers and room temperature water were provided for palate cleansing (Di Renzo et al., 2023). Prior to the sessions, sensory descriptors were established by panel consensus, following ONAV methodology. Panelists rated the intensity (0 = absent to 9 = very intense) of the following attributes: overall judgment, spiciness, herbal notes, acidity, astringency, softness, sweet cherry, red fruits, retro-nasal spiciness, retro-nasal red fruits, and color.

### 2.7 Statistical analyses

Statistical analysis was performed using RStudio (R version 4.3.0). Data from three independent experiments are presented as mean ± standard deviation (SD) and analyzed using ANOVA followed by Tukey’s *post hoc* tests. Statistical significance was considered at *p*-values < 0.05.

## 3 Results

### 3.1 Yeast population dynamics and fermentation performance

**Figure 2:**
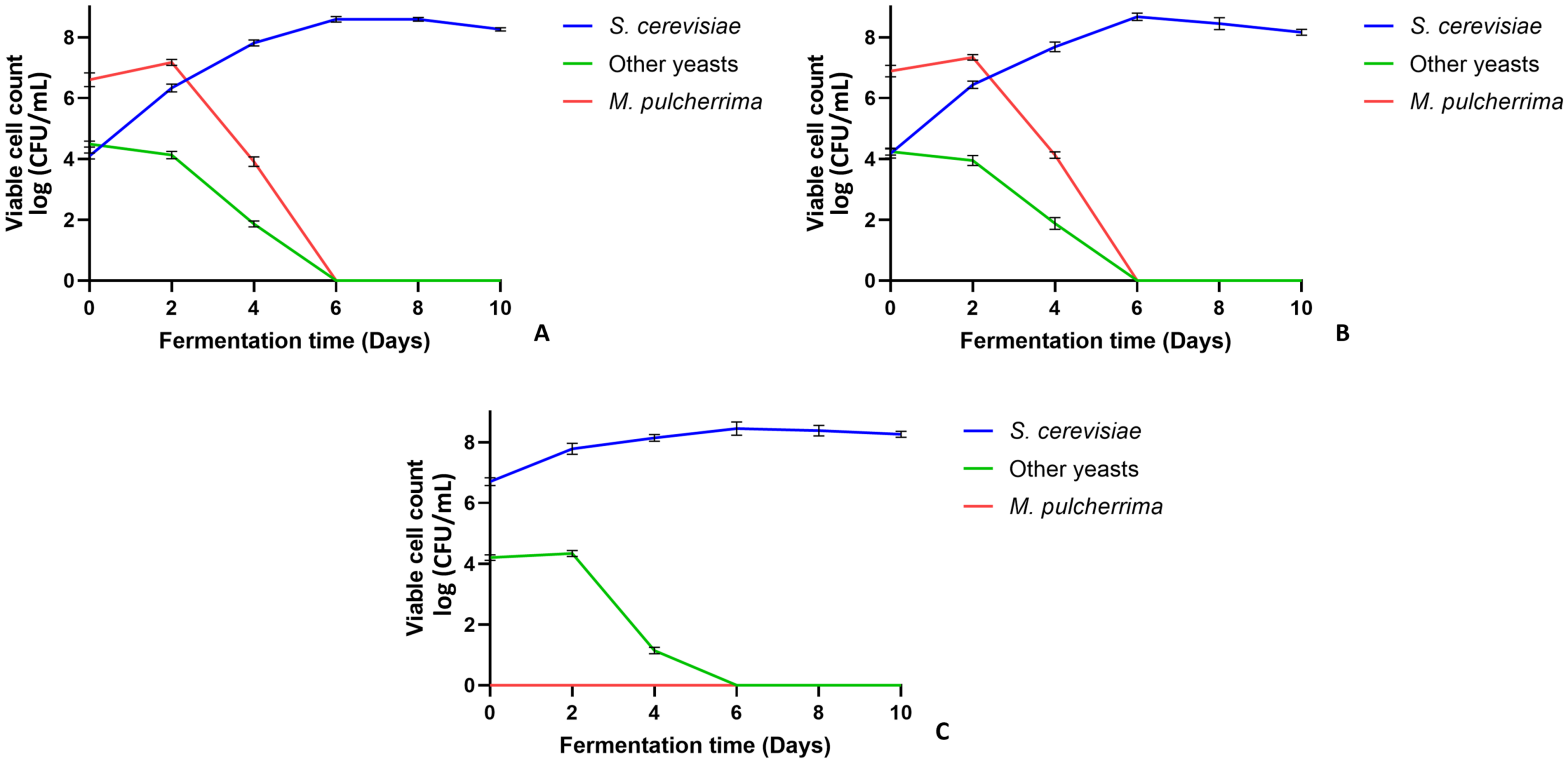
Evolution of yeasts population (log CFU/mL) during the alcoholic fermentation of Kallmet musts. **(A)** *M. pulcherrima* 62 and after 48 h *S. cerevisiae* F15; **(B)** *M. pulcherrima* 62 and after 72 h *S. cerevisiae* F15; **(C)** *S. cerevisiae* F15.

Yeasts dynamics during the fermentation process are shown graphically in Figure 2 and the related numerical data are reported in Supplementary Table S1. In Test A, alcoholic fermentation began with the inoculation of *M. pulcherrima* 62 at a concentration of 6.60 log CFU/mL. The population of *M. pulcherrima* increased to a cell density of 7.17 log CFU/mL after 2 days, then declined to undetectable levels after 6 days of fermentation. *S. cerevisiae* F15, inoculated 48 h after the start of fermentation at 6.33 log CFU/mL, gradually increased and stabilized at approximately 8 log CFU/mL from the sixth day until the end of alcoholic fermentation (10 days). Other non-*Saccharomyces* yeasts were initially present at 4.49 log CFU/mL but rapidly declined to 1.86 log CFU/mL after 4 days, becoming undetectable after 6 days.

In Test B, *M. pulcherrima* 62 was initially inoculated at a concentration of 6.89 log CFU/mL. After 2 days, the cell density of *M. pulcherrima* increased to 7.34 log CFU/mL, then decreased to 4.13 log CFU/mL after 4 days, and subsequently became undetectable.

*S. cerevisiae* F15 was inoculated after 72 h of fermentation at a concentration of 6.00 log CFU/mL, and from the fourth day onward, the cell density remained between 7.68 and 8.16 log CFU/mL. Other non-*Saccharomyces* yeasts were initially present at a concentration of 4.24 log CFU/mL, then rapidly decreased to 1.88 log CFU/mL after 4 days, and became undetectable after 6 days.

In Test C, Kallmet must was inoculated only with *S. cerevisiae* F15 (6.70 log CFU/mL). The density of the *Saccharomyces* yeasts gradually increased, stabilizing from the fourth day onward at approximately 8 CFU/mL until the end of alcoholic fermentation. *M. pulcherrima* was not detected during the entire fermentation phase. Other non-*Saccharomyces* yeasts, initially present in the must at a concentration of 4.20 log CFU/mL, decreased and became undetectable after 6 days.

**Figure 3:**
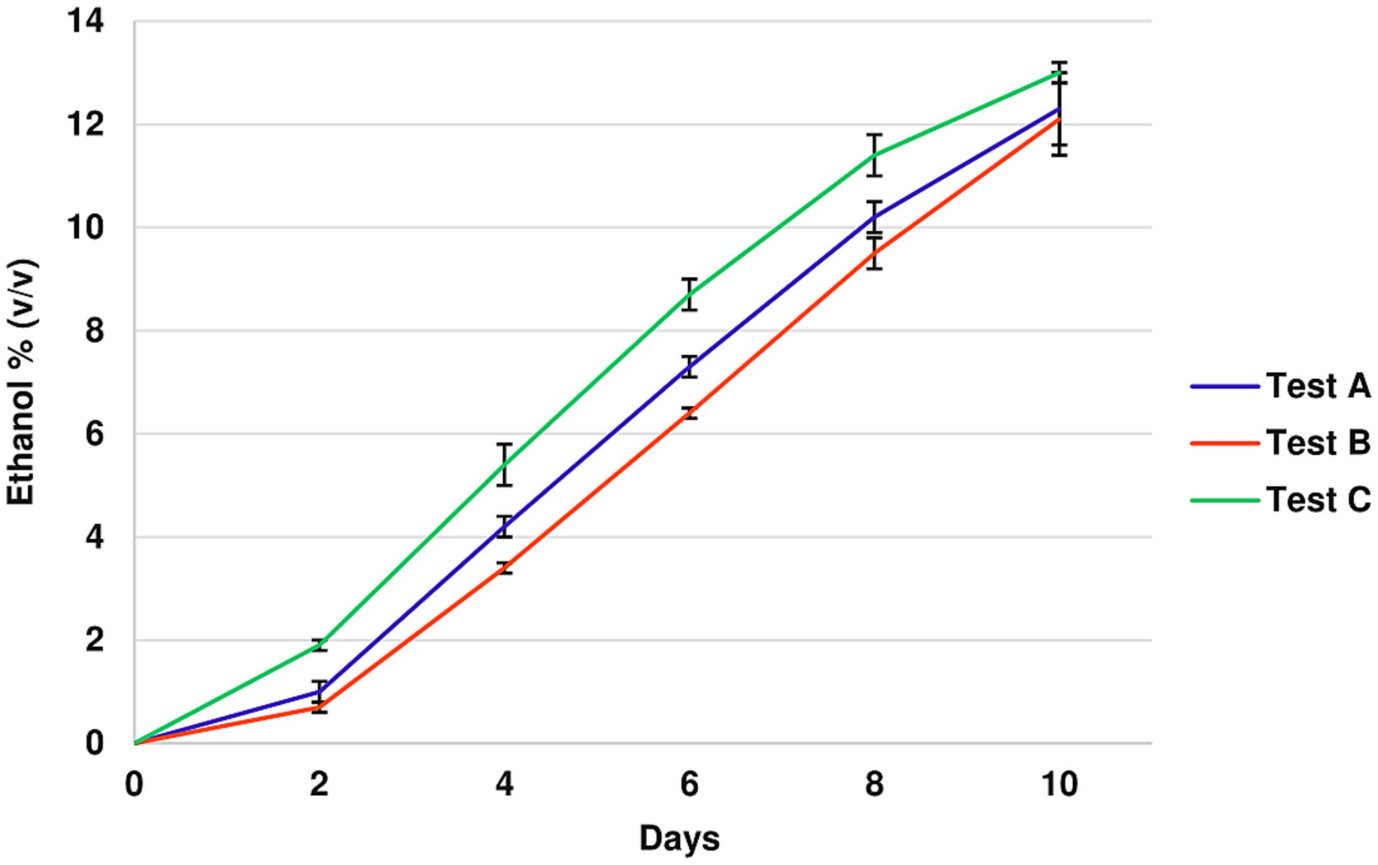
Ethanol evolution (% *v/v*) during the alcoholic fermentation in the different tests. Test A (*M. pulcherrima* 62 and after 48 h *S. cerevisiae* F15); Test B (*M. pulcherrima* 62 and after 72 h *S. cerevisiae* F15) Test C (*S. cerevisiae* F15).

In Test C, where *S. cerevisiae* F15 was initially inoculated, the increase in ethanol concentration was more rapid. However, alcoholic fermentation was completed within 10 days in all trials, as illustrated in Figure 3. The related numerical data are reported in Supplementary Table S2.

### 3.2 Main chemical parameters of wines

The analytical data for the main chemical parameters of the wines are presented in Table 2.

**Table 2:** Main chemical parameters of wines obtained from different fermentation tests.

Chemical parameters	Test A	Test B	Test C
pH	3.6 ± 0.1^a^	3.6 ± 0.1^a^	3.7 ± 0.1^a^
Total acidity (g/L)	6.3 ± 0.5^a^	6.5 ± 0.5^a^	6.6 ± 0.7^a^
Alcohol (% *v/v*)	12.3 ± 0.3^b^	12.0 ± 0.2^b^	13.4 ± 0.3^a^
Volatile acidity (g/L)	0.4 ± 0.1^b^	0.4 ± 0.1^b^	0.7 ± 0.1^a^
L-Malic acid (g/L)	2.1 ± 0.3^a^	1.9 ± 0.1^a^	1.9 ± 0.1^a^
Glycerol (g/L)	6.1 ± 0.2^a^	6.4 ± 0.1^a^	5.3 ± 0.2^b^
Acetaldehyde (mg/L)	5.6 ± 0.3^c^	11.6 ± 0.8^b^	19.3 ± 1.7^a^
Reducing sugars (g/L)	2.1 ± 0.1^a^	2.2 ± 0.1^a^	1.8 ± 0.1^b^
Total polyphenols (mg/L)	1315.0 ± 27.8^b^	1479.3 ± 27.0^a^	1105.0 ± 27.8^c^
Anthocyanins (mg/L)	148.6 ± 5.7^b^	180.7 ± 2.5^a^	131.5 ± 3.4^c^

Test A (M. pulcherrima 62 + S. cerevisiae F15 after 48 h), Test B (M. pulcherrima 62 + S. cerevisiae F15 after 72 h), and Test C (S. cerevisiae F15). Different letters (a-b-c) within a row indicate statistically significant differences (*p* < 0.05).

Sequential inoculation of *S. cerevisiae* at 48 h (Test A) and 72 h (Test B) following the addition of *M. pulcherrima* 62 resulted in wines with lower ethanol contents (12.3 and 12.0% v/v, respectively) than the wine in Test C, which was obtained with must inoculated with *S. cerevisiae* alone (13.4% *v/v*).

No significant differences were observed in pH and total acidity values among the different tests. However, ethanol concentrations differed significantly. In fact, the wine obtained in Test C had the highest alcohol content (13.4% v/v), followed by Test A (12.3% v/v) and Test B (12.0% v/v). Regard the volatile acidity, Tests A and B (both 0.4 g/L) showed a significant decrease compared to Test C (0.7 g/L). L-malic acid concentrations remained similar across all conditions. Glycerol content was highest in Tests A and B (6.1 g/L and 6.4 g/L) and lowest in Test C (5.3 g/L). Acetaldehyde levels differed significantly, with Test C showing the highest concentration (19.3 mg/L), followed by Test B (11.6 mg/L) and Test A (5.6 mg/L). Reducing sugars were comparable in Tests A (2.1 g/L) and B (2.2 g/L), but slightly lower in Test C (1.8 g/L).

Total polyphenol content was significantly higher in Test B (1479.3 mg/L) than in Tests A (1315.0 mg/L) and C (1105.0 mg/L). Similarly, anthocyanin concentrations were highest in Test B (180.7 mg/L), followed by Test A (148.6 mg/L), and lowest in Test C (131.5 mg /L).

### 3.3 Volatile aroma compounds

Table 3 presents the results of the analysis of VOCs detected in the wines. For each compound, the table includes reference data from the literature concerning odor descriptors and the odor thresholds.

**Table 3:** Volatile compounds found in wines obtained using different inoculation strategies.

Higher alcohols (mg/L)	Test A	Test B	Test C	Odor descriptor	Odor threshold (mg/L)	References
2,3-Butanediol	119.6 ± 3.3^a^	**120.7 ± 2.8** ^**a**^	**121.3 ± 2.9** ^**a**^	Butter, creamy	120	López et al. (2004), Li et al. (2008)
Phenylethyl alcohol	**83.2 ± 1.8** ^**b**^	**94.5 ± 1.2** ^**a**^	**39.0 ± 1.5** ^**c**^	Honey, spice, rose, lilac, floral	10	Guth (1997)
Isobutanol	**134.9 ± 2.6** ^**b**^	**170.9 ± 3.1** ^**a**^	**97.6 ± 4.0** ^**c**^	Balsamic, solvent whiskey	40	Guth (1997), Etiévant (2017)
Isoamyl alcohol	**105.5 ± 3.1** ^**b**^	**121.5 ± 3.7** ^**a**^	**60.2 ± 2.1** ^**c**^	Whiskey, malt, burnt	30	Guth (1997)
Methionol	4.9 ± 0.8 ^a^	4.7 ± 0.7 ^a^	3.7 ± 0.2 ^a^	Alcohol, Pungent	9	Peinado et al. (2004)
Hexanol	4.7 ± 0.4 ^a^	5.6 ± 0.4 ^a^	4.6 ± 0.4 ^a^	Ethereal, fruity, alcoholic, sweet, herbaceous	8	Guth (1997)
Butanol	67.3 ± 2.2 ^a^	67.6 ± 2.3 ^a^	66.5 ± 1.5 ^a^	Medicine, fruit	150	Gómez-Míguez et al. (2007)
Pentanol	25.2 ± 1.9 ^a^	25.6 ± 1.4 ^a^	24.5 ± 1.3 ^a^	Balsamic	80	Jiang et al. (2013), Etiévant (2017)
Octanol	0.50 ± 0.14 ^a^	0.55 ± 0.1 ^a^	0.50 ± 0.2 ^a^	Chemical, metal, bunt	0.9	Tao and Zhang (2010)

Terpenes (μg/L)	Test A	Test B	Test C	Odor descriptor	Odor threshold (μg/L)	References
Linalool	**92.2 ± 2.1** ^**b**^	**115.0 ± 4.4** ^**a**^	10.2 ± 1.0^c^	Citrus, floral, woody, green, blueberry	25	Aznar et al. (2003)
Geraniol	82.8 ± 2.6^b^	90.4 ± 3.4^a^	14.0 ± 1.5^c^	Floral, rose, fruity, lemongrass, citrus	130	Čuš and Jenko (2013)
Nerol	94.4 ± 2.9^b^	104.9 ± 4.2^a^	13.1 ± 1.2^c^	Rose-like aromas	400	Čuš and Jenko (2013)

Test A (M. pulcherrima 62 + S. cerevisiae F15 after 48 h), Test B (M. pulcherrima 62 + S. cerevisiae F15 after 72 h), Test C (S. cerevisiae F15). Values exceeding perception threshold levels are shown in bold. Different letters (a-b-c) within a row indicate statistically significant differences (*p* < 0.05).

A total of twelve compounds were identified, including nine higher alcohols and three monoterpenes. It’s particularly notable that both sequential inoculations (Tests A and B) produced significantly higher values than Test C of some VOCs.

In this regard, the highest concentration of phenylethyl alcohol was observed in Test B (94.5 mg/L), followed by Test A (83.2 mg/L) and Test C (39.0 mg/L). Similarly, isobutanol levels were highest in Test B (170.9 mg/L), followed by Test A (134.9 mg/L), and Test C (97.6 mg/L). For isoamyl alcohol, Test B again showed the highest concentration (121.5 mg/L), followed by Test A (105.5 mg/L) and Test C (60.2 mg/L). No significant differences were found for the other higher alcohols analyzed.

Regarding the terpene content in the wines produced, the highest concentrations were detected in Test B (linalool: 115.0 μg/L; geraniol: 90.4 μg/L; nerol: 104.9 μg/L). Slightly lower terpene amounts were observed in Test A (linalool: 92.2 μg/L; geraniol: 82.8 μg/L; nerol: 94.4 μg/L). The lowest terpene concentrations were found in Test C, (linalool: 10.2 μg/L; geraniol: 14.0 μg/L; nerol: 13.1 μg/L).

### 3.4 Sensory evaluation of wines

**Figure 4:**
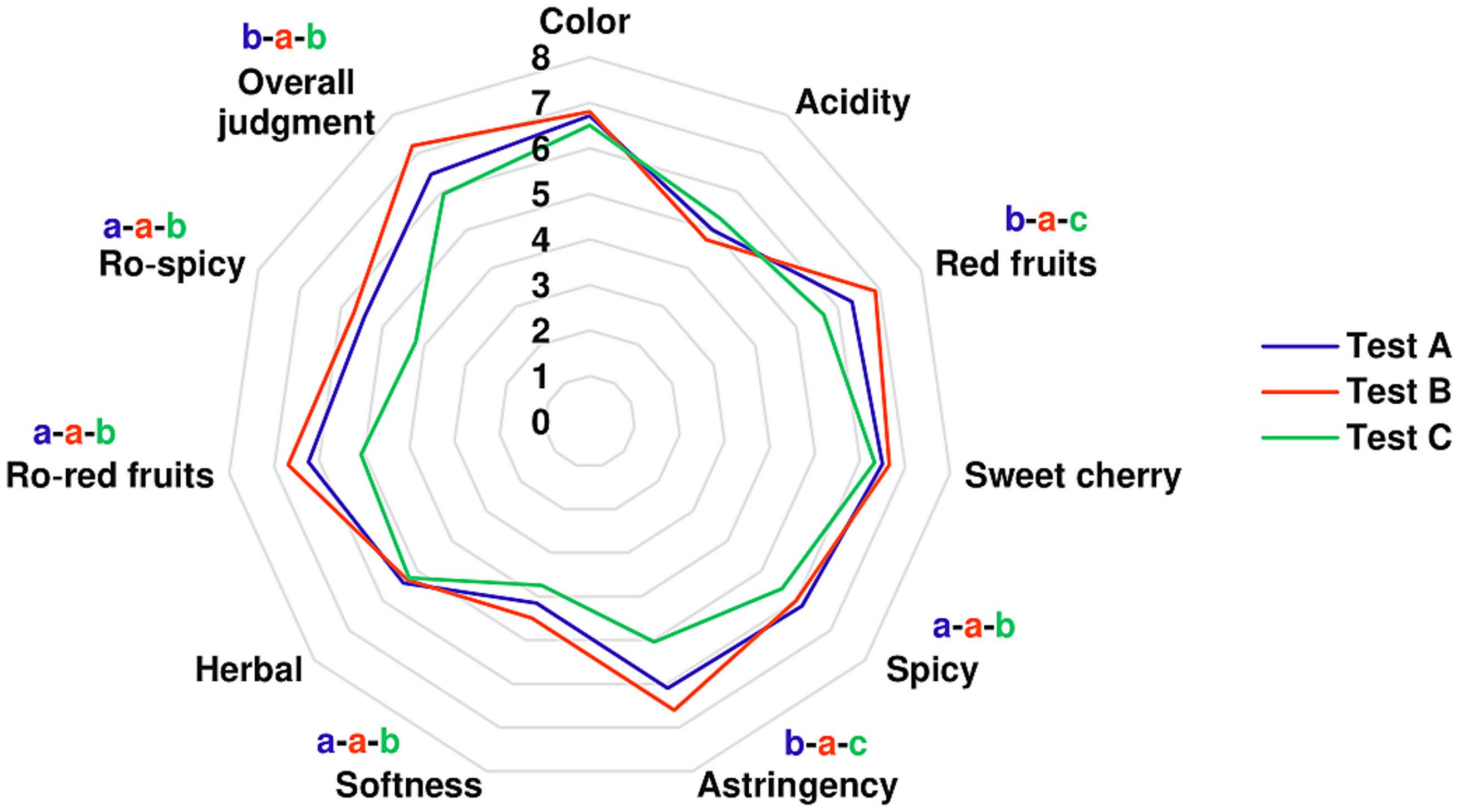
Sensory profiles of Kallmet wines obtained by different fermentation tests: Test A (*M. pulcherrima* 62 + *S. cerevisiae* F15 after 48 h), Test B (*M. pulcherrima* 62 + *S. cerevisiae* F15 after 72 h), and Test C (*S. cerevisiae* F15). Different letters (a-b-c) indicate statistically significant differences (*p* < 0.05).

The results of the sensory analysis of wines are shown graphically in Figure 4 and numerically in Supplementary Table S3. Sensory analysis revealed that the wine obtained in Test B generated the highest overall scores in most of the assessed attributes.

Perceived color intensity was similar across all tests, with no significant differences in scores assigned by the panelists.

The wine obtained in Test B received the highest ratings for red fruit aroma (6.9) and astringency (6.6), significantly outperforming Tests A and C (red fruits: 6.3 and 5.7; astringency: 6.1 and 5.0, respectively). Ratings for sweet cherry and herbaceous notes did not show significant differences between the tests. Test B also scored highest scores for softness (4.5), red fruits aroma (6.9), spiciness (6.0) and ro-spiciness (5.7) while, Test C consistently scored lowest in these attributes. Acidity perception was similar across all tests. The overall judgment was significantly higher for the wine in Test B (7.2), compared to the wines obtained in Test A (6.4) and Test C (5.9).

## 4 Discussion

During alcoholic fermentation, yeast species and their cell density are key factors that greatly influence the compositional and organoleptic characteristics of wine (Zhang et al., 2018a; Canonico et al., 2023). In recent years, increasing attention has been paid to the use of non-*Saccharomyces* yeasts in winemaking as new fermentation starters in combination with *Saccharomyces* yeasts (Jolly et al., 2014). However, several non-*Saccharomyces* yeasts are unable to complete alcoholic fermentation on their own.

In particular, during fermentation, non-*Saccharomyces* yeast species tend to decline due to the competitive advantage of *S. cerevisiae*, which dominates the environment through its efficient sugar metabolism and high alcohol tolerance, thereby limiting the growth and survival of other yeast species (Taillandier et al., 2014; Mencher-Beltrán et al., 2021).

These yeasts show that they are able to persist in the must during the first days of fermentation when the ethanol concentration is still low and are able to express their metabolic potential in these early chronological phases (Fleet, 2003; Wang et al., 2016). The use of sequential inoculation or co-inoculation of non-*Saccharomyces* with *Saccharomyces* yeasts has been proposed as an effective microbiological strategy to simulate spontaneous fermentation and reduce the risk of blocked fermentation, with the aim of improving the organoleptic characteristics of the wine without compromising its compositional quality (Padilla et al., 2016, 2017; Binati et al., 2020; Hranilovic et al., 2020).

Our results showed that in Test A and Test B, the cell density of *M. pulcherrima* decreased from day 4 onwards and became undetectable after 6 days of fermentation, consistent with findings in other studies (Sun et al., 2014; Zhang et al., 2022). However, during these early days, without the nutritional competition from *S. cerevisiae*, *M. pulcherrima* 62 had the opportunity to express its peculiar enzymatic activities.

It should be noted that in Test A and Test B the initial concentration of indigenous *S. cerevisiae* was approximately 10^4^ CFU/mL. These concentrations of indigenous *Saccharomyces*, although relatively high, did not inhibit the growth of *M. pulcherrima* 62. In test A at the time of inoculation of *S. cerevisiae* F15 (48 h) the concentrations of *M. pulcherrima* were approximately 10^7^ CFU/mL. In test B, at the time of inoculation (72 h) of *S. cerevisiae* F15, the populations of *M pulcherrima* and indigenous *S. cerevisiae* were approximately 10^7^ CFU/mL and 10^6^ CFU/mL, respectively. Therefore, both this indigenous population and the commercial starter *S. cerevisiae* F15 may have contributed together with *M. pulcherrima* 62 to the characteristics of the final wine.

Alcoholic fermentation was completed in all tests (Figure 2) within 10 days. Therefore, our results show that *M. pulcherrima* 62 did not negatively interfere with the fermentation kinetics of *S. cerevisiae* during sequential fermentation. These findings are consistent with results obtained in previous studies and show that *M. pulcherrima*, despite having antimicrobial properties against other yeasts and molds, does not inhibit the growth or metabolic activity of *S. cerevisiae* (Oro et al., 2014; Hranilovic et al., 2020; Testa et al., 2024; Coppola et al., 2025).

Sequential inoculations of *M. pulcherrima* 62 and *S. cerevisiae* F15 (Test A and Test B) resulted in wines with lower ethanol content compare to the wine from Test C, which was inoculated solely with *S. cerevisiae*. Previous studies applying sequential inoculations of *M. pulcherrima* and *S. cerevisiae*, have reported ethanol reductions ranging between 0.9 and 2.5% v/v. This variability in alcohol content reduction is likely due to the differences in yeast strain and inoculation protocols (Varela et al., 2017). A study by Hranilovic et al. (2020), emphasized the influence of inoculation timing, showing that inoculating *S. cerevisiae* two days after *M. pulcherrima* resulted in the greatest decrease in ethanol yield.

Given the recent shift in the wine market, the use of non-*Saccharomyces* yeasts such as *M. pulcherrima* could be a promising alternative to reduce the alcohol content in wine (Varela et al., 2017; Testa et al., 2025).

The volatile acidity values (expressed as acetic acid) in all the wines obtained in our study were well within the limits set by current legislation, which specifies a maximum level of 1.2 g/L (Zgardan et al., 2023). Our results are consistent with previous studies that have shown that *M. pulcherrima* has minimal impact on volatile acidity levels in wine (Escribano-Viana et al., 2018). In some cases, the use of *M. pulcherrima* has been linked to reductions in volatile acidity of between 10 and 75%, depending on the strain and fermentation conditions (Hranilovic et al., 2020; Roca-Mesa et al., 2020).

Glycerol is a fermentation by-product in wine production, and it does contribute to enhancing sweetness, smoothness, overall body and wine complexity (Zhao et al., 2015). Our study found that using a sequential inoculum of *M. pulcherrima* with *S. cerevisiae* caused higher glycerol production than using *S. cerevisiae* alone, thus confirming the results obtained by other researchers (Morales et al., 2015; Seguinot et al., 2020). During fermentation with *S. cerevisiae,* increased glycerol production is often associated with elevated levels of acetic acid (Erasmus et al., 2004), while some non-*Saccharomyces* yeasts, such as *M. pulcherrima*, have demonstrated the ability to increase glycerol content without compromising the organoleptic properties of wine by producing excesses of undesirable compounds such as acetic acid (Testa et al., 2024; Coppola et al., 2025).

Acetaldehyde is formed in wine both as an enzymatically derived by-product of yeast metabolism, and as a non-enzymatic oxidation product of ethanol (Danilewicz, 2003).

In our study, the concentration of acetaldehyde found in the wines was very low, particularly in Tests A and B, in which *M. pulcherrima* was used as the initial starter. However, it remained well below the sensory threshold of 100–125 mg/L (Benito et al., 2015) in all tests. Similarly, in other recent studies, it has been shown that in fermentations involving the use of mixed starters (co-inoculum or sequential inoculum) of *M. pulcherrima* and *S. cerevisiae*, lower amounts of acetaldehyde are produced than in fermentations with *S. cerevisiae* in single culture (Muñoz-Redondo et al., 2021; Coppola et al., 2025). At low concentration, acetaldehyde can enhance the fruity aroma of wines and make the ‘green apple’ note more noticeable (Arias-Pérez et al., 2021). However, at higher levels (> 100 mg/L), acetaldehyde can be quite pungent and have a negative impact on wine quality (Garcia et al., 2022).

Regarding the total polyphenol and anthocyanin content, significantly higher concentrations were detected in Tests A and B relative to Test C. Some authors have reported that the use of *M. pulcherrima* in combination with *S. cerevisiae* can improve the color and enhance the polyphenol content, compared to wines fermented with *S. cerevisiae* alone (Belda et al., 2016; Chen et al., 2018; Escribano-Viana et al., 2019). A study by Belda et al. (2016) report that *M. pulcherrima* NS-EM-34 enhances phenolic extraction in winemaking, resulting in increased polyphenol content and improved technological and sensory qualities. This effect is attributed to the production of enzymes that degrade grape cell walls, facilitating the extraction of phenolic compounds during the maceration process.

Yeasts produce higher alcohols through two main pathways: the Ehrlich pathway and *de novo* synthesis from sugars. Both utilize *α*-keto acids as intermediates, which are either derived from amino acid breakdown or de novo synthesis from sugars. In both cases, α-keto acids act as key intermediates, either originating from the transamination or deamination of amino acids, or from sugar metabolism via pyruvate. These α-keto acids are then decarboxylated to aldehydes, which are subsequently reduced by alcohol dehydrogenase to form the corresponding higher alcohols (Styger et al., 2013; Maicas, 2020).

Higher alcohols are volatile compounds that significantly contribute to the aroma profile of wine and play a role in ester biosynthesis (Ferreira, 2011; Kłosowski et al., 2015).

Overall, values below 300 mg/L of higher alcohols provide fruity and floral notes, while values above 400 mg/L become negative because they give wines pungent and unpleasant aromas (Escribano-Viana et al., 2018; Carpena et al., 2020).

It has been well shown that sequential fermentations involving *M. pulcherrima* increase the total concentration of higher alcohols compared to fermentations performed using *S. cerevisiae* alone (Chen et al., 2018; Escribano-Viana et al., 2018). In particular, fermentations involving *M. pulcherrima* often lead to an increase in the concentration of 2-phenylethanol, 1-pentanol, isoamyl alcohol, hexanol, and isobutanol that contribute to the overall aroma and flavor profile of the resulting wine (Rodríguez et al., 2010; Sadoudi et al., 2012; Chen et al., 2018; Escribano-Viana et al., 2018; Dutraive et al., 2019; Seguinot et al., 2020; Kręgiel et al., 2022). In our study, as reported in Table 3, the quantities of some VOCs detected in the wines obtained are higher than those reported in the literature and well above the odor thresholds.

The fermentations carried out with sequential inoculation of *M. pulcherrima* and *S. cerevisiae* (Test A and Test B) showed significantly higher concentrations of isobutanol, phenylethyl alcohol and isoamyl alcohol, compared to those conducted with *S. cerevisiae* alone (Test C).

The amounts of higher alcohols produced during fermentation are significantly influenced by various factors, including fermentation temperature, oxygen and nitrogen availability (Seguinot et al., 2020).

However, yeast strains are the primary determinants of higher alcohol production (Escribano-Viana et al., 2018; Romano et al., 2022). Phenylethyl alcohol, derived from phenylalanine, is characterized by a rose-like aroma (Fang and Qian, 2005) and is consistently associated with positive sensory attributes. In red wines, it has been identified as a major contributor to floral notes, enhancing overall sensory quality (De-La-Fuente-Blanco et al., 2016). Escott et al. (2022) also found that sequential inoculation of *M. pulcherrima* and *S. cerevisiae* during Airén grape fermentation resulted in phenylethyl alcohol concentrations of 30–35 mg/L, compared to 19.7 mg/L in fermentations with *S. cerevisiae* alone.

Isobutanol is a higher alcohol produced during fermentation, primarily via the Ehrlich pathway involving the decarboxylation of amino acids such as valine. In wine, it imparts balsamic, solvent-like, and whisky-like aromas. At concentrations below 300 mg/L, isobutanol is generally considered to contribute positively to the wine aromatic complexity. However, levels exceeding this threshold can impart an unpleasant odour, which may negatively impact the overall sensory profile of wine (De La Fuente Blanco et al., 2017). Regard the isobutanol and isoamyl alcohol, our results are consistent with those of previous studies.

Seguinot et al. (2020) reported that isobutanol concentrations using *S. cerevisiae* in monoculture fermentations ranged from 25 to 62 mg/L. Sequential inoculation with *M. pulcherrima*, however, significantly increased these levels to 99–167 mg/L.

Other studies reported that sequential fermentations with *M. pulcherrima* strains resulted in wine with elevated levels of isoamyl alcohol compared to those fermented solely with *S. cerevisiae*. They also showed, that the co-inoculation of *M. pulcherrima* and *S. cerevisiae* resulted in an increased concentration of isoamyl alcohol and its corresponding ester, isoamyl acetate (Canonico et al., 2023). The intensification of the production of these compounds contributes to creating a distinctive aromatic profile in wines, with isoamyl acetate in particular exerting a strong sensory impact due to its low perception threshold (30 μg/L) and characteristic fruity notes (García et al., 2020; Arias-Pérez et al., 2021).

Terpenes and norisoprenoids are key contributors to the fruity and floral characteristics of wines, as they can be detected by the human nose at extremely low concentrations (Black et al., 2015). In grapes, these aroma-active compounds mainly exist in glycosidically bound, non-volatile forms, in contrast to their free volatile counterparts (Mateo and Jiménez, 2000).

Enzymatic activities of *M. pulcherrima*, particularly *β*-glucosidase and *α*-arabinofuranosidase, play a central role in transforming bound terpenes into their volatile forms that enhance the aroma of wine (Testa et al., 2020; Aplin et al., 2021; Zhang et al., 2021; Perpetuini et al., 2023).

In our study, the wines obtained in Tests A and B exhibited higher concentrations of monoterpenes, such as linalool, geraniol, and nerol, compared to Test C (Table 3). *M. pulcherrima* 62 used in these tests has been shown in previous studies to possess significant β-glucosidase activity (Karaulli et al., 2024), which may explain the increase in free terpene concentrations. Compared to Test C, the highest monoterpene concentrations were found in Tests A and B. These results, as previously discussed and emphasized, are due to sequential fermentations involving *M. pulcherrima* strains compared to single-strain fermentations using only *S. cerevisiae* (Morata et al., 2019; Perpetuini et al., 2023).

These results are consistent with other studies, who reported an increased release of terpenes in Verdicchio and Pecorino wines, when *M. pulcherrima* was used in sequential inoculation with *S. cerevisiae* compared to fermentation with *S. cerevisiae* alone (Canonico et al., 2023; Perpetuini et al., 2023).

Mixed fermentations involving *M. pulcherrima* and *S. cerevisiae* have been shown to significantly increase both the total and individual concentrations of terpenes in other fruit wines as well (Zhang et al., 2022). For example, the sequential inoculation of *M. pulcherrima* and *S. cerevisiae* markedly increased terpene levels in cherry wine (Sun et al., 2014).

In young red wines, anthocyanins are the primary contributors to color. However, during maturation and aging, these compounds undergo reactions that lead to the formation of more complex and stable anthocyanin-derived pigments, resulting in color changes over time (He et al., 2012a, 2012b).

In our study, despite chemical data indicating significant differences in total polyphenol and anthocyanin concentrations (Table 2), there was no perceived difference in color by the judges. While chemical analyses can provide detailed information about wine composition, sensory evaluations, which are based on human perception, may not always align with these findings. This discrepancy highlights the complexity of wine color perception, where factors such as intensity, hue, brightness and clarity play a significant role and may not directly correlate with the chemical composition (Parpinello et al., 2009).

The perception of astringency differed significantly in wine produced in Test C compared to those in Tests A and B. This result is difficult to explain, as astringency is affected by the concentration and interaction of multiple compounds. It is a tactile sensation, mainly caused by the interaction of polyphenolic compounds with salivary proteins. However, the astringency can also be modulated by other constituents such as organic acids, sugars, ethanol, anthocyanins, and polysaccharides (Gawel et al., 2001). In a study by Diako et al. (2016), the impact of polysaccharides on astringency perception was evaluated. They found that polysaccharides can inhibit the interaction between tannins and salivary proteins, thereby reducing astringency perception. Comitini et al. (2011) reported that the final concentration of polysaccharides in wine increased when *M. pulcherrima* was used in mixed fermentation with *S. cerevisiae*.

In our panel test, for the attribute of softness, higher scores were obtained in the wines of Tests A and B than in the wine obtained in Test C. This difference may be partly due to the amount of glycerol produced by *M. pulcherrima*.

Softness is a tactile sensation associated with compounds such as glycerol, higher alcohols and polysaccharides. It is perceived as an enveloping, rounded feeling on the tongue. Polysaccharides play a significant role in the sensory perception of softness. Several studies have demonstrated that non-*Saccharomyces* yeasts have a greater ability to release polysaccharides than *S. cerevisiae* (Giovani et al., 2012; Domizio et al., 2014).

In our study, significant differences were observed in the “spicy” and “red fruit” descriptors, as well as for their retro-olfactive (ro-spicy and ro-red fruit) notes. Specifically, wine from Test C scored very low for spicy notes, compared to Tests A and B, while Test B scored highest for red fruit notes, followed by Tests A and C.

The higher alcohols produced by *M. pulcherrima* during alcoholic fermentation can substantially enhance the fruity and floral aroma profile of red wines. This effect is particularly notable in sequential fermentations, where *M. pulcherrima* contributes to a richer, more complex flavour profile by increasing the levels of higher alcohols, such as phenylethyl alcohol, recognized for its floral character, and other alcohols that intensify fruity aromas (Comitini et al., 2011; Canonico et al., 2023).

Phenylethyl alcohol is a key contributor to floral aromas, which can enhance the sensory quality of wines (De-La-Fuente-Blanco et al., 2016). Notably, the highest concentration was observed when *M. pulcherrima* was allowed to ferment for 72 h prior to the addition of *S. cerevisiae*, confirming that inoculation timing can significantly influence the organoleptic characteristics of the final wine (Tufariello et al., 2012; Ruiz et al., 2019; Coppola et al., 2025).

## 5 Conclusion

Although *M. pulcherrima* is known to possess interesting oenological properties, the few strains available on the market are mainly proposed as biocontrol agents rather than fermentation starters.

This study highlighted the fermentative potential of *M. pulcherrima* 62 when used in sequential inoculation strategies in combination with *S. cerevisiae,* leading to wines with distinct chemical and sensory characteristics. The results showed that sequential inoculation timing is decisive in defining the chemical composition and sensory profile of wines, particularly through its influence on the production of VOCs. Therefore, in addition to the intrinsic metabolic properties of the yeast strain used, it appears extremely important to implement optimized inoculation protocols to promote favorable interactions between yeasts during fermentation. Although preliminary, the data obtained suggest that the use of *M. pulcherrima* in the sequential inoculation with *S. cerevisiae* represent a promising biotechnological approach for the enhancement of quality red wines such as Kallmet. In fact, the enzymatic activities of *M. pulcherrima* 62, in addition to enriching the aromatic component of this wine, have also contributed to increased concentration of total polyphenols and anthocyanins.

Future studies should focus on the application of advanced analytical techniques, such as gas chromatography–mass spectrometry (GC–MS) and high-performance liquid chromatography (HPLC) for profiling of VOCs and phenolic fractions, in order to obtain more detailed data that can provide us with an understanding of interaction of metabolic pathways during fermentation conducted using *M. pulcherrima* 62 as the initial starter in the sequential inoculation with *S. cerevisiae*.

## Data Availability

The original contributions presented in the study are included in the article/Supplementary material, further inquiries can be directed to the corresponding author/s.
