# Supplementary material for: Preliminary insights regarding the quality of Kallmet wine, obtained by sequential inoculation with Metschnikowia pulcherrima and Saccharomyces cerevisiae
Source: Front Microbiol. 2025 Aug 26;16:1654308. doi: 10.3389/fmicb.2025.1654308 (PMC12417457; doi:10.3389/fmicb.2025.1654308)
Supplement: Supplementary file 1 [file Table_1.DOCX]

| **Evolution of yeasts (log CFU/mL) (Days)** | | | | | | | |
| --- | --- | --- | --- | --- | --- | --- | --- |
| **Test** | **Yeasts** | **0** | **2** | **4** | **6** | **8** | **10** |
| **A** | *S. cerevisiae* | 4.10 ± 0.10^e^ | 6.33 ± 0.13^d^ | 7.81 ± 0.10^c^ | 8.59 ± 0.09^a^ | 8.59 ± 0.06^a^ | 8.26 ± 0.05^b^ |
|  | Other yeasts | 4.49 ± 0.10^a^ | 4.13 ± 0.12^b^ | 1.86 ± 0.10^c^ | n.d. | n.d. | n.d. |
|  | *M. pulcherrima* | 6.60 ± 0.23^b^ | 7.17 ± 0.10^a^ | 3.91 ± 0.16^c^ | n.d. | n.d. | n.d. |
| **B** | *S. cerevisiae* | 4.18 ± 0.15^e^ | 6.44 ± 0.12^d^ | 7.68 ± 0.16^c^ | 8.67 ± 0.12^a^ | 8.45 ± 0.20^ab^ | 8.16 ± 0.10^b^ |
|  | Other yeasts | 4.24 ± 0.11^a^ | 3.95 ± 0.17^b^ | 1.88 ± 0.20^c^ | n.d. | n.d. | n.d. |
|  | *M. pulcherrima* | 6.89 ± 0.19^b^ | 7.34 ± 0.09^a^ | 4.13 ± 0.11^c^ | n.d. | n.d. | n.d. |
| **C** | *S. cerevisiae* | 6.70 ± 0.13^c^ | 7.78 ± 0.18^b^ | 8.14 ± 0.11^ab^ | 8.45 ± 0.22^a^ | 8.38 ± 0.17^a^ | 8.26 ± 0.10^a^ |
|  | Other yeasts | 4.20 ± 0.09^a^ | 4.34 ± 0.10^a^ | 1.14 ± 0.11^b^ | n.d. | n.d. | n.d. |
|  | *M. pulcherrima* | n.d. | n.d. | n.d. | n.d. | n.d. | n.d. |

**Table S1.** Evolution of yeasts (log CFU/mL) during the alcoholic fermentation of the Kallmet musts. Test A (*M. pulcherrima* 62 + *S. cerevisiae* F15 after 48 h), Test B (*M. pulcherrima* 62 + *S. cerevisiae* F15 after 72 h), Test C (*S. cerevisiae* F15). Different letters (a-b) within a row indicate significant differences (p < 0.05). n.d. (not detected) represents a value below the detection limit (1 log CFU/g).
