# Supplementary material for: Preliminary insights regarding the quality of Kallmet wine, obtained by sequential inoculation with Metschnikowia pulcherrima and Saccharomyces cerevisiae
Source: Front Microbiol. 2025 Aug 26;16:1654308. doi: 10.3389/fmicb.2025.1654308 (PMC12417457; doi:10.3389/fmicb.2025.1654308)
Supplement: Supplementary file 2 [file Table_2.DOCX]

| **Time (days)** | **A** | **B** | **C** |
| --- | --- | --- | --- |
| 0 | 0 ± 0 | 0 ± 0 | 0 ± 0 |
| 2 | 1.0 ± 0.2^b^ | 0.7 ± 0.1^b^ | 1.9 ± 0.1^a^ |
| 4 | 4.2 ± 0.2^b^ | 3.4 ± 0.1^c^ | 5.4 ± 0.4^a^ |
| 6 | 7.3 ± 0.2^b^ | 6.4 ± 0.1^c^ | 8.7 ± 0.3^a^ |
| 8 | 10.2 ± 0.3^b^ | 9.5 ± 0.3^b^ | 11.4 ± 0.4^a^ |
| 10 | 12.3 ± 0.3^b^ | 12.0 ± 0.2^b^ | 13.4 ± 0.3^a^ |

**Table S2**. Ethanol evolution (% *v/v*) during the alcoholic fermentation in the different tests. Test A (*M. pulcherrima* 62 + *S. cerevisiae* F15 after 48 h), Test B (*M. pulcherrima* 62 + *S. cerevisiae* F15 after 72 h), Test C (*S. cerevisiae* F15). Different letters (a-b) within a row indicate significant differences (p < 0.05).
