# Supplementary material for: Preliminary insights regarding the quality of Kallmet wine, obtained by sequential inoculation with Metschnikowia pulcherrima and Saccharomyces cerevisiae
Source: Front Microbiol. 2025 Aug 26;16:1654308. doi: 10.3389/fmicb.2025.1654308 (PMC12417457; doi:10.3389/fmicb.2025.1654308)
Supplement: Supplementary file 3 [file Table_3.DOCX]

**Table S3.** Sensory profiles of Kallmet wines obtained by different fermentation tests: Test A (*M. pulcherrima* 62 + *S. cerevisiae* F15 after 48 h), Test B (*M. pulcherrima* 62 + *S. cerevisiae* F15 after 72 h), and Test C (*S. cerevisiae* F15). Different letters (a-b) within a row indicate significant differences (p < 0.05).

| **Sensory perception** | **Test A** | **Test B** | **Test C** |
| --- | --- | --- | --- |
| Color | 6.7 ±0.51^a^ | 6.8 ±0.10^a^ | 6.5 ±0.30^a^ |
| Acidity | 5.0 ±0.31^a^ | 4.7 ±0.27^a^ | 5.3 ±0.15^a^ |
| Red fruits | 6.3 ±0.19^b^ | 6.9 ±0.23^a^ | 5.7 ±0.28^c^ |
| Sweet cherry | 6.5 ±0.40^a^ | 6.6 ±0.35^a^ | 6.3 ±0.42^a^ |
| Spicy | 6.2 ±0.17^a^ | 6.0 ±0.15^a^ | 5.6 ±0.10^b^ |
| Astringency | 6.1 ±0.20^b^ | 6.6 ±0.20^a^ | 5.0 ±0.14^c^ |
| Softness | 4.1 ±0.35^a^ | 4.5 ±0.39^a^ | 3.7 ±0.15^b^ |
| Herbal | 5.4 ±0.47^a^ | 5.3 ±0.17^a^ | 5.2 ±0.32^a^ |
| Ro‐red fruits | 6.2 ±0.34^a^ | 6.7 ±0.21^a^ | 5.1 ±0.27^b^ |
| Ro‐spicy | 5.4 ±0.35^a^ | 5.7 ±0.20^a^ | 4.2 ±0.30^b^ |
| Overall judgment | 6.4 ±0.30^b^ | 7.2 ±0.25^a^ | 5.9 ±0.14^b^ |
